# Anesthetic drugs modulate feeding behavior and hypothalamic expression of the POMC polypeptide precursor and the NPY neuropeptide

**DOI:** 10.1186/s12871-018-0557-x

**Published:** 2018-07-27

**Authors:** E. Besnier, T. Clavier, M. C. Tonon, G. Pelletier, B. Dureuil, H. Castel, V. Compère

**Affiliations:** 1 Normandie Univ, UNIROUEN, INSERM U1239, DC2N, 76000 Rouen, France; 2 Institute for Research and Innovation in Biomedicine (IRIB), 76000 Rouen, France; 3 Normandie Univ, UNIROUEN, INSERM U1096, EnVi, 76000 Rouen, France; 4 Department of Anesthesiology and Critical Care, Rouen University Hospital, Rouen, France; 5 Research Center in Molecular Endocrinology, Oncology and Genetics, Laval University Hospital Center, Quebec, G1V4G2, Canada

**Keywords:** Anesthetics, Feeding behavior, Neuropeptide Y, Pro-opiomelanocortin, Arcuate nucleus

## Abstract

**Background:**

Several hypnotic drugs have been previously identified as modulators of food intake, but exact mechanisms remain unknown. Feeding behavior implicates several neuronal populations in the hypothalamic arcuate nucleus including orexigenic neuropeptide Y and anorexigenic pro-opiomelanocortin producing neurons. The aim of this study was to investigate in mice the impact of different hypnotic drugs on food consumption and neuropeptide Y or pro-opiomelanocortine mRNA expression level in the hypothalamic arcuate nucleus.

**Methods:**

Saline control, isoflurane, thiopental, midazolam or propofol were administered to C57Bl/6 mice. Feeding behavior was evaluated during 6 h. In situ hybridization of neuropeptide Y and pro-opiomelanocortine mRNAs in the hypothalamus brain region was also performed. Data were analyzed by Kruskal Wallis test and analysis of variance (*p* < 0.05).

**Results:**

Midazolam, thiopental and propofol induced feeding behavior. Midazolam and thiopental increased neuropeptide Y mRNA level (respectively by 106 and 125%, *p* < 0.001) compared with control. Propofol and midazolam decreased pro-opiomelanocortine mRNA level by 31% (*p* < 0,01) compared with control. Isoflurane increased pro-opiomelanocortine mRNA level by 40% compared with control.

**Conclusion:**

In our murine model, most hypnotics induced food consumption. The hypnotic-induced regulation of neuropeptide Y and pro-opiomelanocortine hypothalamic peptides is associated with this finding. Our data suggest that administration of some hypnotic drugs may affect hypothalamic peptide precursor and neuropeptide expression and concomittantly modulate food intake. Thus, this questions the choice of anesthetics for better care management of patients undergoing major surgery or at risk of undernutrition.

**Electronic supplementary material:**

The online version of this article (10.1186/s12871-018-0557-x) contains supplementary material, which is available to authorized users.

## Background

Early postoperative oral food intake has been suggested to decrease post-surgical complications and reduce length of hospital stay [1, 2]. Several factors may promote postoperative oral feeding such as analgesia, multimodal anti-emetic treatment and enforced oral nutrition [3]. Another factor that can typically interfere with early recovery of oral food intake is the postoperative appetite impairment.

Hypothalamic neuropeptide signaling systems play an important role in the control of food intake and energy expenditure in mammals [4]. These systems include two interconnected populations of neurons located in the arcuate nucleus (ARC), one producing the orexigenic neuropeptide Y (NPY) and the other the anorexigenic peptide α-melanocyte-stimulating hormone, a processing product of the pro-opiomelanocortin (POMC) precursor [4–6].

Anesthetic drugs modulate appetite in some studies. Propofol, midazolam and phenobarbital have been shown to increase food intake, whereas the administration of isoflurane has been shown to reduce feeling of hunger in comparison with propofol in one human study [7–12]. Nevertheless, the effect of these drugs has not been compared within a study and their effects on hypothalamic gene expression levels have not yet been reported. Therefore, in the present study, we examined the role of various hypnotics on food intake and on the hypothalamic mRNA levels encoding peptides involved in the control of appetite (hypothalamic NPY and POMC mRNA levels).

## Methods

### Animals

Male C57BL/6 mice were obtained from Charles River Laboratories (St. Constant, QC, Canada) of 12–14 weeks old housed at a constant temperature (21 °C) and under a 14 h/10 h light/dark cycle (lights on 6 a.m.). All animals had free access to standard chow (proteins 21.4%, lipids 5.1%, fibers 4%, mineral ashes 5.4%, moistures 12.1% and nitrogen free extracts 52%; A03 diet, SAFE corp France) and drinking tap water. All protocols used in this study were approved by our local ethic committee. All procedures were performed in accordance with the French Ethics Committee as well as the guidelines of the European Parliament directive 2010/63/EU and the Council for the Protection of Animals Used for Scientific Purposes under the supervision of an authorized investigator. The elaboration of this manuscript adheres to the ARRIVE (Animal Research: Reporting of In Vivo Experiments) guidelines.

### Food intake measurement

Five groups of 6 C57B/l6 mice were constituted to explore the effects of various hypnotic drugs on food consumption. Since the main objective was to test the orexigenic effect of some drugs, we realized the experiments during the period of least activity, *i.e* daytime period, where feeding behavior is normally reduced. At 9 a.m. animals received an intraperitoneal injection of NaCl 0.9% (control saline) or anesthetic drug thiopental (50 mg/kg, Panpharma© France) or sedative drug midazolam (5 mg/kg, Panpharma© France) or intravenous administration of propofol (10 mg/kg, B Braun© Medical France) or isoflurane 2.5% inhaled during 3 min (Baxter© France). In order to assess the hypnotic effects of anesthetics, we measured the time until recovery of righting reflex after administration in 6 additional mice per group. One hour after administration, isolated mice in individual cages were presented with 2.6 g of their usual food. No water was available during the duration of the experiment. To avoid hiding food, cages were devoid of litter during the duration of the experiment. Hourly ingestat was determined by regular weighing of food during 6 h after administration of the hypnotic drug. Ingestat was expressed in gram (g).

### In situ hybridization

#### Experimental groups and materials

Five groups of 8 animals were also constituted to explore the impact of anesthetics on mRNA expression. One hour before experimentation, the animals were submitted to a protocol similar to the one used for in vivo food intake experiment: intraperitoneal injection of NaCl 0.9% (control saline) or midazolam (5 mg/kg) or thiopental (50 mg/kg) or intravenous administration of propofol (10 mg/kg) or isoflurane 2.5% inhaled during 3 min.

#### Tissue preparation

For histological purposes, mice were deeply anesthetized (ketamine-xylazine) and then immediately perfused transcardially with 4% paraformaldehyde in 0.2 M phosphate buffer between 8 and 10 a.m. and exactly one hour after drug administration. Brains were removed and postfixed in the same fixative overnight at 4 °C, and then placed in 15% sucrose in 0.1 M phosphate buffer overnight at 4 °C. Thereafter, the tissues were frozen on dry ice in support medium (OCT, Bayer Corp., Elkhart, IN, USA). The sections were serially cut at 10 μm with a cryostat, then mounted on Superfrost/PLUS Microscope slides (Fisher Scientific, Montreal, Canada) and maintained at − 80 °C until use.

#### Hybridization

In situ hybridization was performed as described previously [13]. Specific antisens oligonucleotide probe for POMC and NPY were used as described previously [13, 14]. Probes were labeled with 35S-adATP using terminal deoxynucleotidyl transferase. After hybridization, the brain sections were dehydrated and coated with liquid photographic emulsion (Kodak NTB-2). They were processed after 7 days of exposure.

Semi-quantitative analysis of hybridization signal was carried out on nuclear emulsion-dipped slides over the confines of reactive cells in the ARC of the hypothalamus using Zeiss Optical System coupled with a computer and Mercator Image Software (PRIMACEN platform). The optical density (OD) of the hybridization signal was measured under dark-field illumination at 10× magnification. Sections from control and treated animals were matched for rostrocaudal level. The hypothalamus was digitized and subjected to densitometric analysis with ImageJ software (version 1.44p, NIH, USA), yielding measurements of integrated OD. The OD of each specific region was then corrected for the average background signal, which was determined by sampling tissues immediately outside the cell group of interest. The operator was blinded to the anesthetic drug used in each group.

### Statistical analysis

In the in situ hybridization study, quantitative data are presented as mean values with a confidence interval of 95%. Comparison of mRNA levels between experimental groups was performed by analysis of variance (ANOVA) followed by Dunnett’s multiple comparison test. In oral food intake measurements, data are presented as mean values with a confidence interval of 95%. Comparisons of duration of anesthesia and ingestat were performed by Kruskal-Wallis test followed by Dunn’s multiple comparison test. Each analysis was performed with GraphPad Prism v6.0 software (La Jolla California USA). For both analyses, comparisons were performed between NaCl 0.9% control and anesthetics. *P* < 0.05 was considered statistically significant.

## Results

### Food intake

**Fig. 1:**
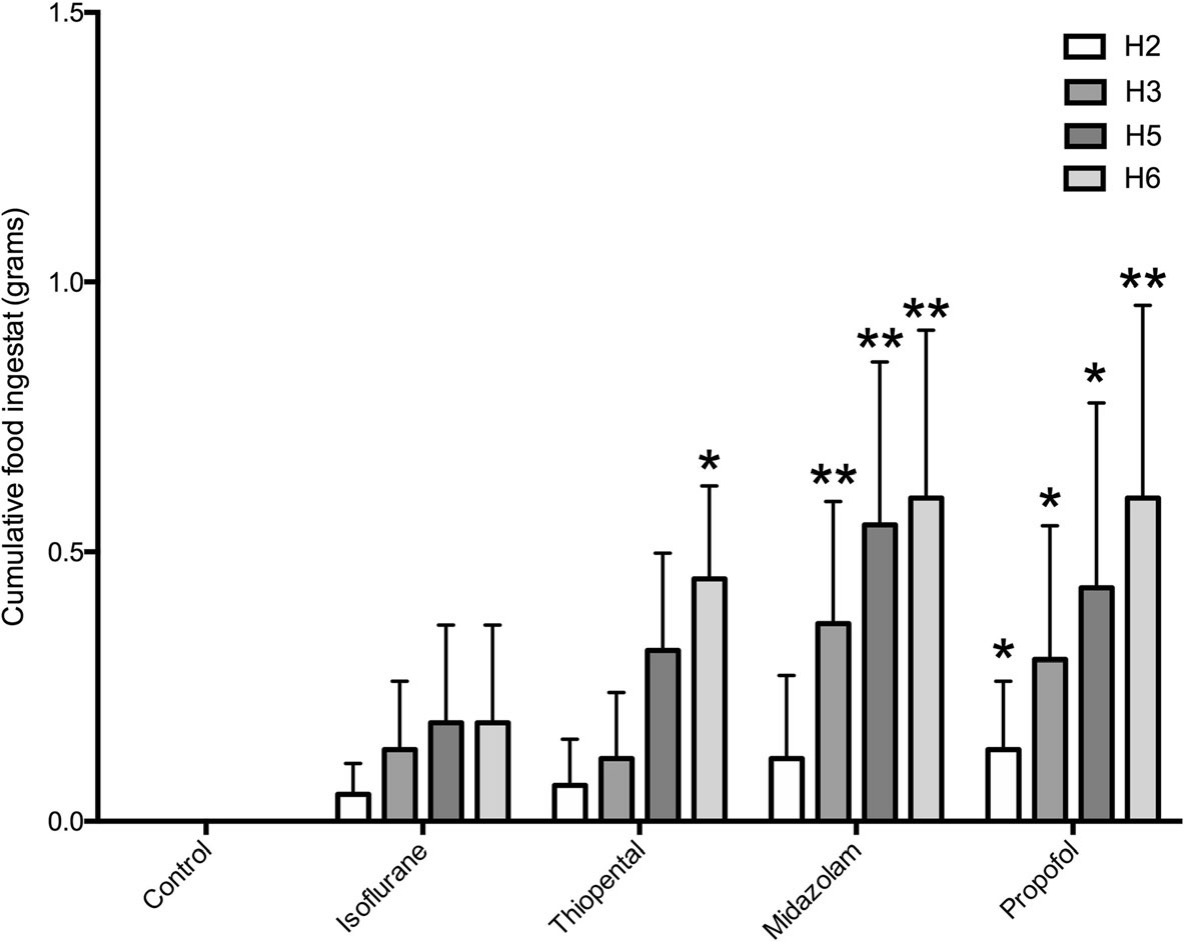
Impact of various hypnotic drugs on cumulative oral food intake from 2 to 6 h after their administration in mice compared with NaCl 0.9% control group. Results are expressed in grams and presented as means with confidence interval of 95% (*n* = 6 mice per group). ** *P* < 0.01, * *P* < 0.05, NaCl control versus other groups at each time. Comparisons has been made using Kruskal-Wallis test

After administration of isoflurane, propofol and thiopental, mice presented a rapid loss of righting reflex. Recovery from anesthesia first occurred in the isoflurane group (48 [41–55] seconds), then the propofol group (258 [235–282] seconds) and finally in the thiopental group (1687 [675–2699] seconds). The only significant difference was observed between thiopental and isoflurane group (global ANOVA *p* < 0.0001, Dunn’s multiple comparison *p* = 0.0003, Additional file 1). Midazolam administration was not associated with a loss of righting reflex but only a small diminution in motor activity. In all groups a complete recovery was observed within 1 h after anesthetic administration. Figure 1 presents results for the amount of ingestat after drug administration. No ingestat was noted in the control group receiving NaCl according to the expected feeding cycle of mice. Mice receiving propofol presented a significant increased food intake from 2 to 6 h after drugs administration. Those with midazolam showed a significant increased food intake from 3 to 6 h after administration. A significant increase in food intake after thiopental administration was observed at 6 h. Isoflurane administration did not result in a significant increase in food intake.

### Hypothalamic NPY mRNA expression

**Fig. 2:**
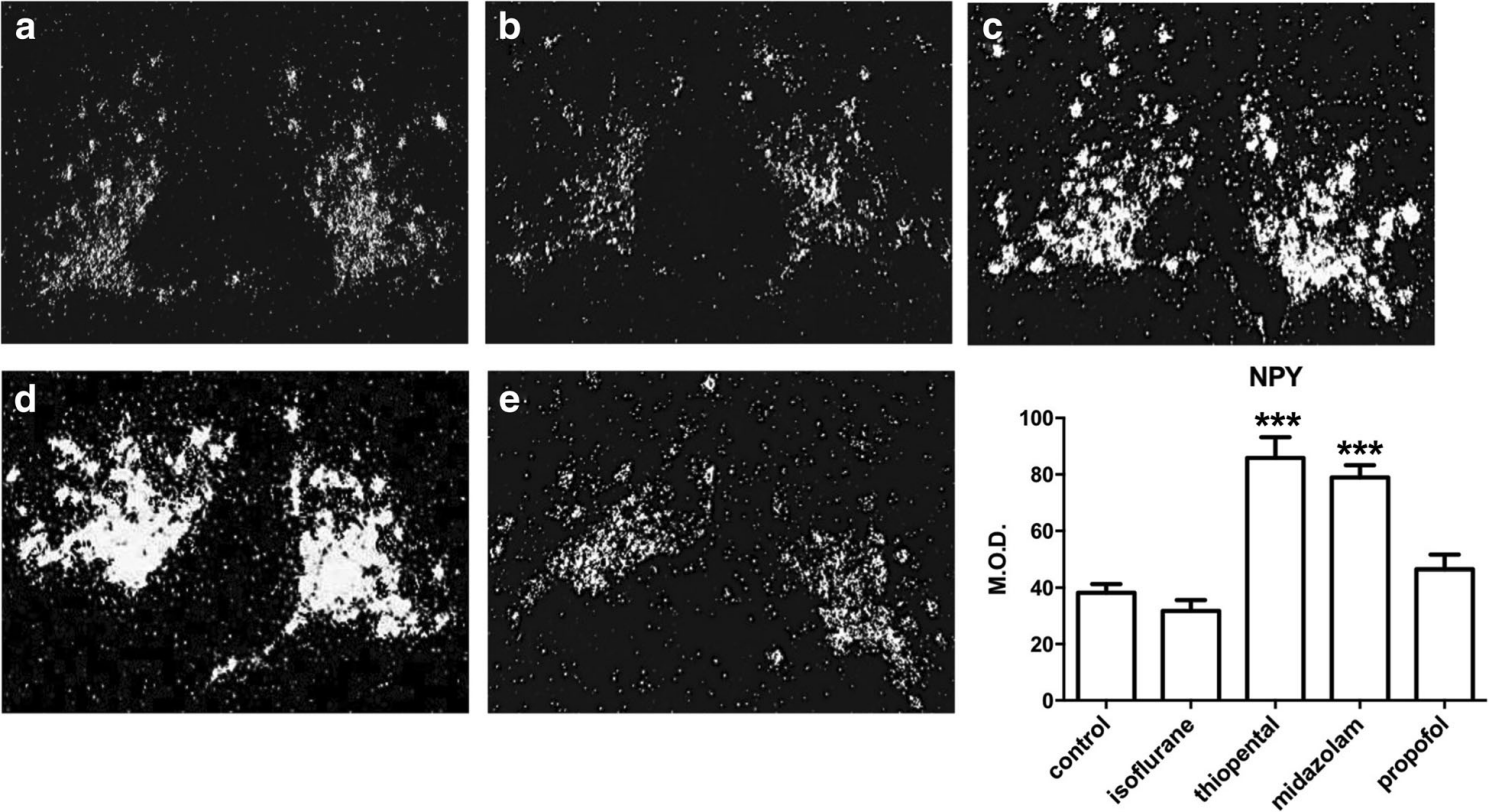
NPY mRNA level expression evaluated by quantification of In Situ Hybridization after hypnotics administration. X10 magnification darkfield microphotographies illustrate the expression of NPY mRNA in hypothalamus. **a** control, (**b**) isoflurane, (**c**) thiopental, (**d**) midazolam, (**e**) propofol. Results are also presented as means with confidence interval of 95% (*n* = 8 mice per group). *** *P* < 0.001 NaCl control versus other groups. NPY: Neuropeptide Y, M.O.D.: Mean Optical Density. Comparisons has been made using an Analysis of Variance

Labeling obtained after in situ hybridization with the [35S]-NPY encoding RNA probe was essentially distributed throughout the ARC of the hypothalamus, with high intensity of signal found in the area surrounding the third ventricle. The analysis of variance presents a *p* value < 0.0001. Administration of isoflurane or propofol did not significantly change NPY mRNA expression. Administration of midazolam was associated with a 106% increase in NPY expression when compared with saline-injected animals (*p* < 0.001). Administration of thiopental also increased by 125% NPY mRNA levels compared with control (*p* < 0.001) (Table 1, Fig. 2).

**Table 1:** mRNA level expression of NPY and POMC in different treated groups

	NPY	POMC
Control	38.22 [35.11-41.32]	16.42 [14.61-18.23]
Isoflurane	31.82 [28.05-35.59]	23 [21.25-24.74]***
Thiopental	85.91 [78.64-93.18]***	18.99 [16.86-21.12]
Midazolam	78.93 [74.6-83.25]***	11.31 [9.24-13.37]**
Propofol	46.47 [41.3-51.64]	11.23 [9.061-13.4]**

Data are presented as means with 95% confidence intervals. NPY: Neuropeptide Y, POMC: pro-opiomelanocortin. * *p*<0.05, ** *p*<0.01, ***p<0.001 versus control group. ANOVA *p* value for both experiments are <0.0001

### Hypothalamic POMC mRNA expression

**Fig. 3:**
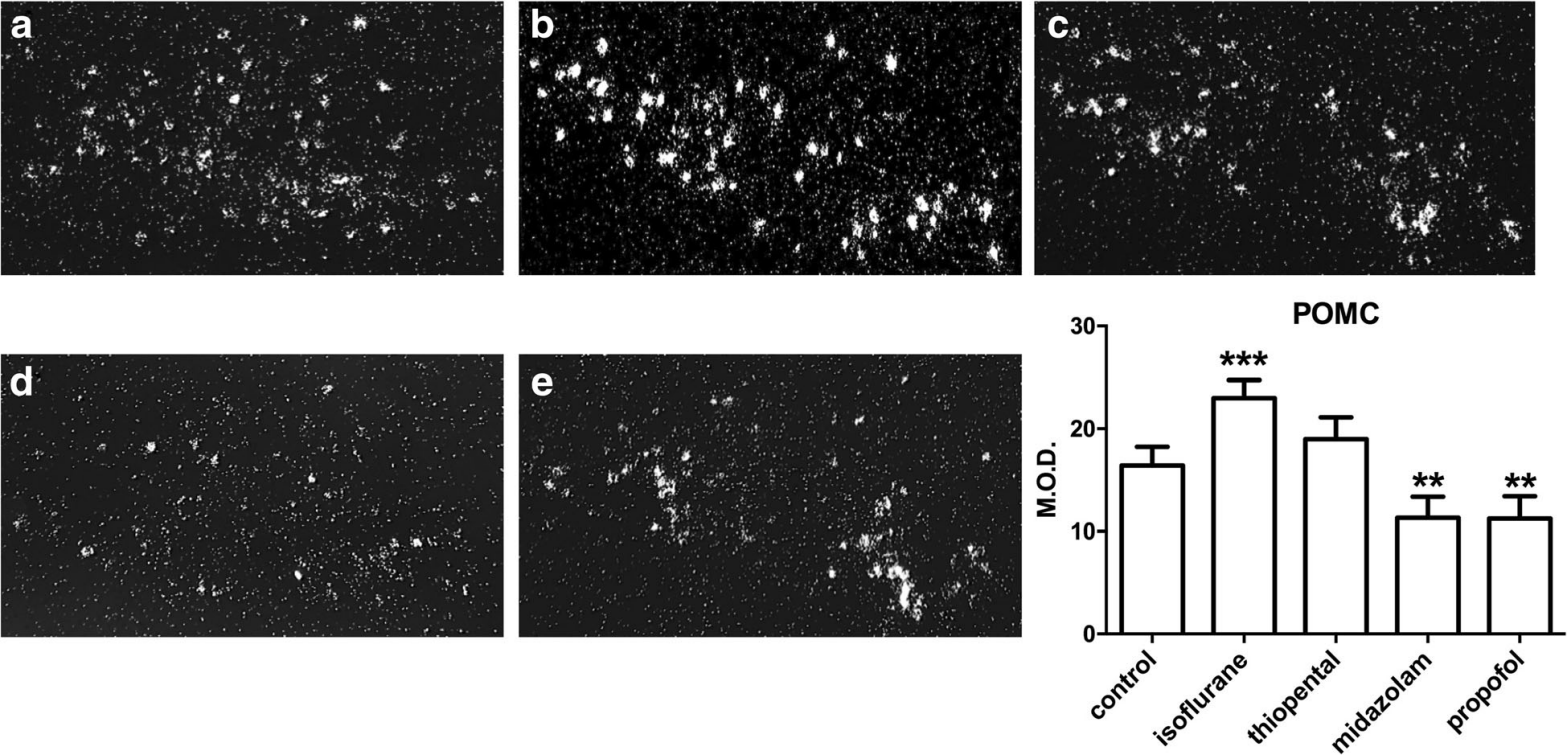
POMC mRNA level expression evaluated by quantification of In Situ Hybridization after hypnotics administration. X10 magnification darkfield microphotographies illustrate the expression of POMC mRNA in the hypothalamus. **a** control, (**b**) isoflurane, (**c**) thiopental, (**d**) midazolam, (**e**) propofol. Results are also presented as means with confidence interval of 95% (*n =* 8 mice per group). ** *P <* 0.01, *** *P <* 0.001 NaCl control versus other groups. POMC: pro-opiomelanocortin. M.O.D.: Mean Optical Density. Comparisons has been made using an Analysis of Variance

POMC labeled antisens RNA probe showed a large distribution in the ARC. As illustrated by light microscope dark-field microphotographies, neurons expressing POMC mRNA were mostly observed in the outer area of the ARC. The analysis of variance presents a *p* value < 0.0001. Administration of midazolam and propofol induced a decrease in POMC mRNA level by 31% (*p* < 0.01) and 32% (*p* < 0.01) respectively, compared with the control saline group. Conversely, isoflurane produced a POMC mRNA level increase of 40% compared with control (*p* < 0.001) (Table 1, Fig. 3).

## Discussion

Our original study describes some effects of systemic administration of anesthetic drugs on hypothalamic arcute nucleus peptide mRNA expression, in association with modified feeding behavior. However, hypnotic-induced effects depend on the drug tested. Some results of this study deserve comments. The lack of randomization and the potential for bias must be taken into account. The choice of hypnotic doses used in our work was based on the doses known to induce anesthetic effects in mice [15]. All anesthetics used induced similar hypnotic effects on mice with comparable recovery time, likely after 30 min.

We observed increased appetite in mice following midazolam administration. Benzodiazepines, to which midazolam belongs, are drugs currently used for anxiolytic or sedative effects and have been shown to stimulate appetite in both rats and humans independently from anxiolytic effect [9, 10, 12]. They exert their action through potentiation of the activity of the γ-aminobutyric acid type A receptor (GABA_A_R) [16], which is known to promote feeding behavior. Indeed, Stratford et al. showed that micro-infusion of muscimol (a GABA_A_R agonist) near lateral ventricules and into the nucleus accumbens shell, induced strong feeding behavior in rats [17]. In addition, it has been demonstrated that administration of flumazenil, a specific antagonist of the benzodiazepine site on the GABA_A_R, abolishes hyperphagic response to intracerebroventricular administration of midazolam [10]. This suggests that the orexigenic effect of midazolam can be mediated by the GABA_A_R. Accordingly, hypothalamic POMC and NPY neurons are both able to synthesize GABA and GABA_A_Rs, which appear to be present in both neuronal populations [18–20]. Our study showed that midazolam inversely modifies peptide mRNA expression with a rise in NPY and a decrease in POMC expression. These data are in agreement with a previous report suggesting that benzodiazepine drugs inhibit POMC gene expression in the rat ARC [21, 22].

Propofol also exerts anesthesia through stimulation of the GABA_A_R [16]. Furthermore, it has also been shown to stimulate appetite and feelings of hunger in the post-operative recovery period in humans [7, 8, 11, 23]. Our results are in agreement with this observation because propofol administration markedly favored appetite in mice. This finding could be related to a decrease in anorexigenic POMC mRNA level in ARC. However, no effect was observed on NPY mRNA level, as previously observed in a clinical study by Grouzmann et al. (2000) in which propofol administration did not change NPY mRNA level in the cerebroventricular fluid during craniotomy [7] but induced effective appetite in patients. Therefore, it is possible to hypothesize that the orexigenic effect of propofol could be related to inhibition of POMC hypothalamic anorexic pathway rather than NPY orexigenic stimulation.

Isoflurane is a soluble volatile GABAergic anesthetic currently used in general anesthesia [24]. Currently no study to evaluate the effect of this drug on food intake can be found. Nevertheless, data comparing propofol with volatile anesthetics have highlighted altered food intake by volatile anesthetics compared with anesthesia using propofol [7]. Our study shows that isoflurane did not significantly stimulate food intake and further induced a rise in mRNA level encoding POMC, known to be anorexigenic. Currently, no data could explain this result. The anesthetic effect of several drugs are mediated by GABA_A_R exhibiting different subunit compositions depending on neuronal population or brain area [16]. The hypothalamic neuronal population are equiped by different subtypes of GABA_A_R [24]. This suggests the anesthetic GABA_A_R agonist could modulate food intake depending on subunits composition in some specific neuronal populations of ARC.

Thiopental also exerts anesthesia through the GABA_A_R [16]. Thiopental is known to induce an increase in food intake in rats at similar dosages to those in our study, but to a lesser extent than benzodiazepines [12]. These results are in agreement with our data and may be related to a stimulation of the orexigenic NPY pathway.

Our data show that some drugs used during anesthesia exert changes in feeding behavior and this effect is associated with modulation of two interconnected populations of neurons (POMC and NPY) in hypothalamic ARC. One hypothesis that could explain these observations would be a direct hypothalamic stimulation by anesthetic drugs. The very short time between anesthetic administration and observed modulation of mRNA expression in ARC (less than one hour) supports this hypothesis of direct modulation. Thus, the orexignic effect observed in mice could be due, according to the anesthetic used, to stimulation of expression of NPY (by thiopental) or to a decrease in the expression of POMC (by propofol) or both (by midazolam). The absence of effect for halogenated volatile drug isoflurane could be due to the stimulation of expression of POMC in ARC. The discrepancy between halogenated and other drugs could be explained by different affinities for specific subunits composition of hypothalamic GABA_A_R. Indeed, the GABA_A_R is composed of 5 glycoproteic subunits. Several subtypes of subunits exist resulting in multiple possible compositions, each presenting a specific sensitivity to the different anesthetics. Thus, isoflurane effects are highly dependent from α subunit and this agent has an increased sensitivity to receptors comprising an α5 or α6 subunits; benzodiazepine effects are mainly dependent on α1 and α2 subunits; and intravenous propofol and thiopental effects appear to be primarily subject to the type of β subunit (for a complete review see [25]). On the other hand, there is heterogeneity in the expression of the GABA_A_R subunits within the brain, particularly in the hypothalamic areas including the NPY and POMC neurons of the ARC [19]. Indeed, POMC neurons contain α 1, α2 and α3 subunits and NPY neurons contain α3 subunits, which could explain the effects of midazolam, while the presence of α5 subunits in the ventromedian nucleus of the hypothalamus, containing orexin receptors with neuronal projections to the ARC, could explain the effects of isoflurane [19, 26]. Thus, the variable effect of halogenated drugs compared with other anesthetics could be related to this heterogeneity within key areas of food intake regulation. However, to date, no study has evaluated the in vivo binding of hypnotics to the subunits of the GABA_A_R in the various areas of the brain to support or refute this hypothesis.

Although the ARC is the main centre for food intake regulation, other brain areas influence this function. Indeed, NPY and POMC neurons present numerous projections in adjacent areas such as the paraventricular nucleus, producing cortisol releasing hormone with anorexigenic proprieties, and the lateral hypothalamus producing orexin A and B with orexigenic proprieties. In particular, it has been demonstrated that activation of GABA_A_R by a specific agonist (muscimol) in the accumbens nucleus induced activation of the orexin-producing neurons of the lateral hypothalamus and a decrease in the activity of the POMC neurons in the ARC, resulting in an increase in food intake [27] . On the other hand, there is a social and environmental component to food intake. Thus, the limbic system and its reward system is involved in the regulation of food intake, in particular related to galanin-producing neurons, which themselves present projections towards the NPY neurons of the ARC. This neuropeptide has orexigenic properties with a stimulation of NPY production. For complete reviews see [28, 29]. All these data demonstrate the complexity and interconnections between systems, making it difficult to identify a specific target for the effects of anesthetics. However, it appears that the ARC is at the crossroads of these systems and the presence of GABA_A_Rs make it a potential target for anesthetics. Another hypothesis for the effects observed in our study could be an indirect pathway of anesthetic with modulation of systemic factors involved in feeding behavior, such as ghrelin, insulin or leptin, which could, at a later stage, modulate the neuronal activity in ARC.

Despite these interesting results, transposition to humans, especially in a surgical context, is difficult. Indeed, anesthesia in surgical context is not limited to the use of a single anesthetic but to a combination, inducing complex and combined effects on many physiological variables, and therefore probably on the feeding behavior. In addition, surgical stress and pain are identified as altering the feeling of hunger. If our work does not provide full certainty on the choice of anesthetic, it deepens our understanding of the effects of gabaergic drugs on the complex system of food intake and opens up therapeutic possibilities for post-operative rehabilitation. Thus, we are awaiting the results of a randomized controlled study on the effects of propofol versus sevoflurane on post-operative feeling of hunger, which may provide additional elements of response (NCT02272166).

## Conclusion

In conclusion, our study deepens the knowledge of the effect of various gabaergic anesthetics on food intake regulation. Depsite the transposition to humans is premature, it could open up therapeutic prospects. One parameter leading to the choice of the anesthetic drug may be determined by their effect on the food intake following general anesthesia. It would allow a diminution of starvation time after surgery, with particular interest after some surgery in which rapid recovery of feeding is important, such as colorectal surgery, thus influencing prognosis. This understanding of the impact of anesthetics on orexigenic behavior, could find wider application in patients including those suffering from cancer, anorexia nervosa or other etiologies. Another area of application could be patients undergoing ambulatory surgery where rapid feeding recovery would be not only a source of comfort but also shorter hospital stay. Thus, in all situations where early recovery of feeding is beneficial to patients, use of drugs such as propofol may be preferred to use of halogenated anesthetics. Nevertheless, further clinical studies must be performed to confirm the human in vivo effect of anesthetic drugs on the control of food intake.

## Additional files

Additional file 1: Recovery from anesthesia evaluated by the recovery of the righting reflex, in seconds, after admininstration of the different anesthetics. *n* = 6 per group. (JPG 299 kb)

Additional file 2: Database of measured values. (XLSX 16 kb)

## Abbreviations

ANOVA: Analysis of variance
ARC: Arcuate nucleus
ARRIVE: Animal Research: Reporting of In Vivo Experiments guidelines
GABA: γ-Aminobutyric acid
GABA_A_R: γ-aminobutyric acid type A receptor
mRNA: Messenger ribonucleotid acid
NPY: Neuropeptide Y
OD: Optical density
POMC: Pro-opiomelanocortine
