# Supplementary figures and images for: Anesthetic drugs modulate feeding behavior and hypothalamic expression of the POMC polypeptide precursor and the NPY neuropeptide
Source: BMC Anesthesiol. 2018 Jul 27;18:96. doi: 10.1186/s12871-018-0557-x (PMC6064126; doi:10.1186/s12871-018-0557-x)

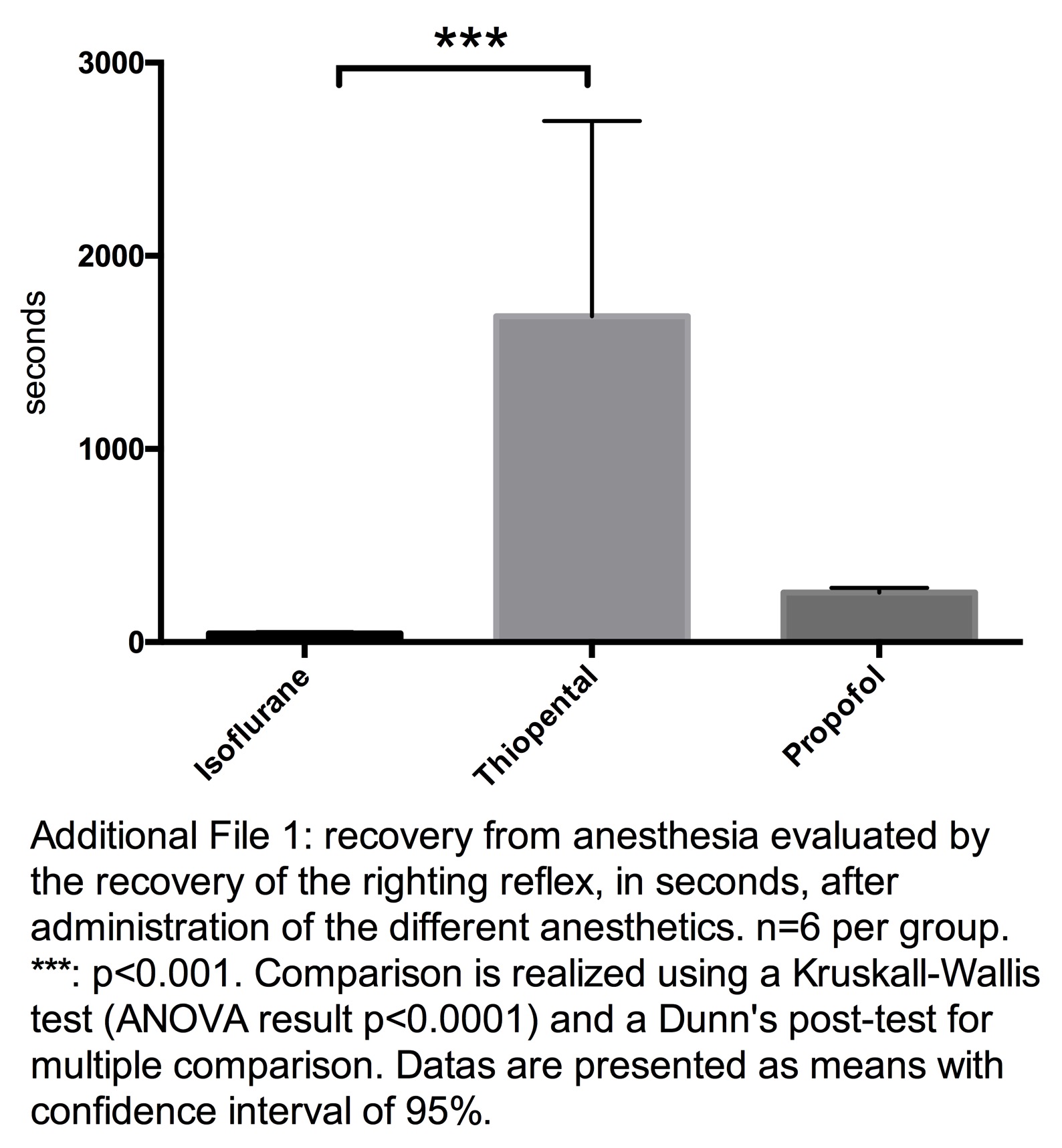

Supplement: Supplementary file 1 — Recovery from anesthesia evaluated by the recovery of the righting reflex, in seconds, after admininstration of the different anesthetics. n = 6 per group. (JPG 299 kb) [file 12871_2018_557_MOESM1_ESM.jpg]
